# Psychiatric comorbidities on an inpatient dermatology consultation service: A cross‐sectional analysis

**DOI:** 10.1002/ski2.266

**Published:** 2023-07-13

**Authors:** Thomas Norman, Jana Guenther, Natalia Vecerek, Brandon L. Adler, Ashley Crew, Scott Worswick

**Affiliations:** ^1^ Keck School of Medicine University of Southern California Los Angeles California USA; ^2^ Department of Dermatology Keck School of Medicine University of Southern California Los Angeles California USA

## Abstract

Despite the high prevalence of psychiatric illness in hospitalised dermatology patients, characterisation of psychiatric comorbidities on an inpatient dermatology consultation service in the United States has yet to be performed. To fill this gap in knowledge, we investigated the prevalence of and factors associated with psychiatric illness on the inpatient dermatology consultation service at the University of Southern California. Of the 429 patients seen by the dermatology consultation service between June 2021 to July 2022, 147 (34%) had psychiatric illness (defined as having at least 1 psychiatric diagnosis). Increasing age was associated with a decreased likelihood of psychiatric illness, while housing instability, chronic dermatologic disease, drug reaction, and pruritus without rash were associated with an increased likelihood of psychiatric illness. The high prevalence of psychiatric illness observed in hospitalised dermatology patients emphasises the importance of collaboration between consultant dermatologists and mental health specialists, particularly when specific sociodemographic or disease factors are present.

Dear Editor,

Despite the high prevalence of psychiatric illness in dermatology patients,^1^ a characterisation of psychiatric comorbidities on an inpatient dermatology consultation service in the United States has yet to be reported. This gap in knowledge could lead to underrecognition of psychiatric illness when confronted with the diverse range of diagnoses observed in the hospital setting.

We determined the prevalence of and factors associated with psychiatric illness in hospitalised dermatology patients at the University of Southern California, which includes 2 private hospitals and 1 public safety net hospital. We retrospectively identified adults seen by the inpatient dermatology consultation service between June 2021 and July 2022, recording age, gender, ethnicity/race, insurance, hospital, housing status, and Charlson Comorbidity Index (CCI). CCI predicts long‐term mortality based on existing medical conditions^2^ and was used to approximate physical disease burden. We also recorded the dermatologic diagnosis, chronicity, and whether it was the reason for admission. Dermatologic diagnoses were divided into 11 groups (Table 1). Dermatologic disease was defined as acute (symptoms <6 months) or chronic (≥6 months). Psychiatric diagnoses were based on International Classification of Diseases, version 10 codes and primary team documentation. Patients were divided into those with psychiatric illness (≥1 psychiatric diagnosis) and those without psychiatric illness (no psychiatric diagnoses).

**TABLE 1:** Description of patient characteristics according to presence of psychiatric illness.

Factor	Study cohort (*n* = 429)	No psychiatric illness (*n* = 283)	Psychiatric illness (*n* = 146)	*P*‐value^a^
Age, years	53.1 ± 16.2	55.8 ± 16.5	47.7 ± 14.1	<0.001
Ethnicity/race^b^				<0.001
Asian or Pacific Islander	44 (10)	34 (12)	10 (7)	
Hispanic	220 (51)	158 (56)	62 (42)	
Non‐hispanic black	39 (9)	16 (6)	23 (16)	
Non‐hispanic white	65 (15)	41 (14)	24 (16)	
Housing status				<0.001
Stably housed	378 (88)	267 (94)	111 (76)	
Unstably housed	51 (12)	16 (6)	35 (24)	
Charlson comorbidity index				0.03
0–1	108 (25)	61 (22)	47 (32)	
2–3	117 (27)	76 (27)	41 (28)	
4+	204 (48)	146 (52)	58 (40)	
Dermatologic disease chronicity^c^				0.006
Acute (<6 months)	312 (73)	219 (77)	93 (64)	
Chronic (≥6 months)	111 (26)	62 (22)	49 (34)	
Dermatologic diagnostic class^d^				0.009
Infection	97 (23)	62 (22)	35 (24)	
Drug reaction	65 (15)	33 (12)	32 (22)	
Vascular	59 (14)	43 (15)	16 (11)	
Psoriasis or dermatitis	39 (9)	27 (10)	12 (8)	
Cutaneous malignancy	28 (7)	22 (8)	6 (4)	
Vesiculobullous disease	20 (5)	15 (5)	5 (3)	
Connective tissue disease	17 (4)	12 (4)	5 (3)	
Pruritus without rash	17 (4)	6 (2)	11 (7)	
Trauma‐induced	16 (4)	10 (4)	6 (4)	
Undifferentiated	24 (6)	21 (7)	3 (2)	
Other	47 (11)	32 (11)	15 (10)	

*Note*: Data are mean ± standard deviation or number (%). Only factors significant on *t*‐test or chi‐square tests are shown.

^a^ Welch's unequal variance *t*‐test was used for age. Chi‐square test was used for all other comparisons.

^b^ 61 patients were identified as either “other” or “unknown” in their electronic medical record.

^c^ 6 patients had unknown disease chronicity.

^d^ “Undifferentiated” referred to patients without a leading diagnosis. “Other” included patients who had a likely diagnosis that did not fit into the other categories.

Characteristic differences between groups were analysed using *t*‐test and chi‐square test. Univariable and multivariable logistic regressions were performed to identify factors associated with psychiatric illness. Since our cohort included patients with drug reactions caused by illicit substances and psychiatric medications, these patients contributed to the association between drug reactions and psychiatric illness. Therefore, a sensitivity analysis was conducted, whereby multivariable analysis was repeated after excluding these patients. *P* < 0.05 was considered statistically significant. R (version 4.2.2) was used. This study was approved by our institutional review board (HS‐18000640).

In total, 429 adults were seen by our inpatient dermatology consultation service (Table 1). Patients were most frequently male (258 [60%]), Hispanic (220 [51%]), covered by government insurance for low‐income individuals (235 [55%]), seen at the public hospital (265 [62%]), and stably housed (378 [88%]). Dermatologic disease was usually acute (312 [73%]). It represented the primary reason for hospitalisation in less than half of instances (190 [44%]). The most common diagnostic categories were infection, drug reaction, and vascular disorder. Of the 429 patients, 146 (34%) had ≥1 psychiatric diagnosis, most commonly substance use disorder (89 [21%]), major depressive disorder (43 [10%]), generalised anxiety disorder (28 [7%]), and schizophrenia, bipolar, or schizoaffective disorder (17 [4%]). Among patients with psychiatric illness, 21 (14%) were evaluated by psychiatry during the current hospitalisation.

On multivariable regression, increasing age was associated with a lower likelihood of psychiatric illness (odds ratio [OR], 0.97; 95% confidence interval [CI], 0.94–0.98; *P* < 0.001). Factors associated with a higher likelihood of psychiatric illness were housing instability (OR, 5.22; 95% CI, 2.52–10.81; *P* < 0.001), chronic dermatologic disease (OR, 2.17; 95% CI, 1.22–3.83; *P* = 0.008), drug reaction (OR, 5.22; 95% CI, 1.66–16.39; *P* = 0.005), and pruritus without rash (OR, 7.90; 95% CI, 1.86–33.58; *P* = 0.005). Drug reaction was attributed to a psychiatric medication or an illicit substance in 6 patients. On sensitivity analysis, after removing these patients, drug reactions remained associated with psychiatric illness (OR, 4.46; 95% CI, 1.40–14.16; *P* = 0.011).

In other countries, the prevalence of psychiatric illness in hospitalised dermatology patients has been as high as 38%.^1^ This is comparable to the 34% observed in our patients, although the cited study reported on inpatients at a specialised dermatologic hospital, rather than those seen by a consultation service.

The higher likelihood of psychiatric illness with housing instability, dermatologic disease chronicity, and pruritus without rash are consistent with the literature. Although the association between psychiatric illness and all drug reactions has not been previously reported, prior studies have demonstrated connections between psychiatric disease and specific drug reactions, including after Stevens‐Johnson syndrome/toxic epidermal necrolysis.^3^ The lack of association between psoriasis/dermatitis and psychiatric illness could be partially explained by aggregating multiple diseases to form this group. Nationally, the prevalence of comorbid psychiatric illness is 38.7% for psoriasis^4^ versus 16.36% for atopic dermatitis.^5^

The lower likelihood of psychiatric illness with increasing age could reflect that individuals with mental health disorders die a median of 10 years earlier than the general population.^6^ While unnatural causes of death are higher in individuals with psychiatric illness, the main source of death in this population is from natural causes (eg medical comorbidities), suggesting that lifestyle, social determinants, and barriers to care may also contribute to the survival disparity.^6^

This study was limited by retrospective nature, small sample size, and single‐institution origin. Nevertheless, the high prevalence of psychiatric illness observed in hospitalised dermatology patients emphasises the importance of collaborating with mental health specialists. Future research is warranted to determine how the observed prevalence compares to that seen by consultation services of other specialties. Additionally, while it has been demonstrated that comorbid psychiatric illness increases length of hospitalisation^7^ and rate of readmission,^8^ it is necessary to determine whether these worse outcomes are seen in dermatology inpatients specifically.

## CONFLICT OF INTEREST STATEMENT

Brandon L. Adler has served as a research investigator and/or consultant to AbbVie and Skin Research Institute, LLC.

## AUTHOR CONTRIBUTIONS

**Thomas Norman**: Conceptualization (equal); data curation (lead); formal analysis (lead); investigation (equal); methodology (equal); visualization (equal); writing—original draft (lead); writing—review & editing (equal). **Jana Guenther**: Conceptualization (equal); data curation (supporting); formal analysis (supporting); investigation (equal); methodology (equal); writing—original draft (supporting); writing—review & editing (equal). **Natalia Vecerek**: Conceptualization (equal); data curation (supporting); investigation (equal); writing—original draft (supporting); writing—review & editing (equal). **Brandon L Adler**: Conceptualization (equal); data curation (supporting); investigation (equal); supervision (supporting); writing—original draft (supporting); writing—review & editing (equal). **Ashley Crew**: Conceptualization (equal); data curation (supporting); investigation (equal); supervision (supporting); writing—original draft (supporting); writing—review & editing (equal). **Scott Worswick**: Conceptualization (equal); data curation (supporting); formal analysis (supporting); investigation (equal); methodology (equal); project administration (equal); supervision (lead); visualization (equal); writing—original draft (supporting); writing—review & editing (equal).

## FUNDING INFORMATION

This article received no specific grant from any funding agency in the public, commercial, or not‐for‐profit sectors.

## ETHICS STATEMENT

Study was approved by our IRB. HS‐18‐00640.

## Data Availability

The data that support the findings of this study are available from the corresponding author upon reasonable request.
